# Prevalence and characteristics of amblyopia, strabismus, and refractive errors among patients aged 3–16 years in Shanghai, China: a hospital-based population study

**DOI:** 10.1186/s12886-024-03477-8

**Published:** 2024-06-07

**Authors:** Jiali Wu, Ning Wang

**Affiliations:** Department of Ophthalmology, Shanghai General Hospital, School of Medicine, Shanghai Jiao Tong University, Wujin Road No.85, Hongkou district, Shanghai, 200080 China

**Keywords:** Visual impairment, Amblyopia, Strabismus, Refractive error

## Abstract

**Background:**

Functional visual impairments in children are primarily caused by amblyopia or strabismus. This study aimed to determine the prevalence and clinical profile of amblyopia and strabismus among individuals aged 3–16 years in Shanghai, China.

**Methods:**

From February 2023 to February 2024, this hospital-based, cross-sectional study included data of children who visited the Ophthalmology Department of Shanghai General Hospital. Comprehensive ocular examinations included visual acuity measurement after cycloplegic refraction, slit lamp examination, cover test, and dilated fundus examination. Descriptive statistics were performed to estimate the proportion and clinical characteristics of amblyopia and strabismus.

**Results:**

A total of 920 children were enrolled in our study. Among them, 223 (24.24%) children were identified as amblyopia. Unilateral amblyopia occupied 57.85%, and bilateral amblyopia occupied 42.15%. Most participants were within the age range of 5–10 years (75.97% for unilateral amblyopia, and 70.21% for bilateral amblyopia). Anisometropia was the primary cause of unilateral amblyopia (68.99%). Most amblyopic children have high hyperopia (38.76% for unilateral amblyopia, and 39.89% for bilateral amblyopia). 30 (3.26%) children were diagnosed with strabismus, and 19 (63.3%) of them were aged 5–10 years. Seven of the children had both strabismus and amblyopia.

**Conclusion:**

The proportion of patients with amblyopia and strabismus was determined as 24.24% and 3.26% in our study. Anisometropia was the leading cause of unilateral amblyopia, whereas high hyperopia was a crucial refractive error in the amblyopic population. These findings shed light on further longitudinal studies targeting the age-related changes in amblyopia, strabismus and refraction errors. Therefore, efforts should be made to manage uncorrected refractive errors, amblyopia, and strabismus among children in Shanghai.

**Supplementary Information:**

The online version contains supplementary material available at 10.1186/s12886-024-03477-8.

## Background

The World Health Organization (WHO) reported that visual impairment (VI) is a major health problem in many countries, with approximately 19 million children and adolescents aged 5–15 years suffering from VI. Amblyopia and strabismus are the two main causes of paediatric VI, with an estimated incidence of 1–3.5% worldwide. Amblyopia is defined as reduced visual acuity (VA) accompanied by many factors, such as anisometropia, strabismus, and vision deprivation [1]. Mainstream treatments include appropriate diopter correction, patching or surgery. However, 15–50% of amblyopic children fail to achieve normal VA, mainly due to the late modalities [2]. Strabismus is another common ocular disorder that leads to VI, and contributes significantly to amblyopia. The estimated prevalence of concomitant strabismus is 2.3–6% [3]. Notably, the long-term surgical successes for strabismus were unsatisfactory (72.67% for horizontal strabismus, i.e.) [4]. Therefore, early screening for amblyopia and strabismus is imperative to ensure timely intervention.

Previous studies have identified the prevalence of VI among children in Shanghai, a major centre of economic activity in China. The reported rates of amblyopia ranged widely from 0.93 to 10.12% of children suffering from VI. Apart from amblyopia, rare studies evaluated the prevalence of strabismus among children in Shanghai. A study carried out in Nanjing estimated a prevalence of 5.56% of strabismus [5]. These findings underline the necessity for an in-depth investigation of the epidemiological data and clinical profiles of amblyopia and strabismus in Shanghai.

Our study aimed to assess the prevalence and further identify the clinical pattern of amblyopia and strabismus among patients aged 3–16 years old in Shanghai, China.

## Methods

### Ethics statement and study design

This study was a hospital-based, cross-sectional study. All the procedures were performed in accordance with the Declaration of Helsinki principles. All experimental protocols were approved by Institutional Review Board Committee of Shanghai General Hospital, Shanghai JiaoTong University (Approval No.IIT2023-087). All the enrolled participants in this study went to Ophthalmology department of Shanghai General Hospital, Shanghai, China, from February 2023 to February 2024. Informed consent was obtained from all subjects and/or their legal guardian(s).

### Examinations and definitions

Demographic data were collected. Comprehensive ocular examinations included best-corrected visual acuity (BCVA) measurement after cycloplegic refraction, slit-lamp examination, alternate prism and cover test, axial length (AL) (IOL Master 500; Carl Zeiss Meditec AG, Jena, Germany), and dilated fundus examination. Amblyopia diagnosis was based on the preferred pattern by the American Academy of Ophthalmology in 2017. Spherical equivalent (SE) was calculated by adding the spherical power and half the magnitude of the cylinder power.

#### Definition

1. Refractive error:

Hyperopia: ≥+1.5 dioptres (D). 1).Mild:≤+2.0 D; 2).Moderate:+2.25 ~ + 5.0 D; 3).High:>+5.0 D. Myopia: ≥-0.75 D. 1).Mild:≤-3.0 D; 2).Moderate:-3.25~-6.0 D; 3). High:>-6.0 D. Myopia: ≥-0.75 D. 1).Mild:≤-3.0 D; 2).Moderate:-3.25~-6.0 D; 3). High:>-6.0 D.

2. Strabismus

A unilateral cover/uncover test was performed with a distant picture fixation target and a near figure puppet, with and/or without spectacles. Any movement of the uncovered eye after occlusion of the test eye for 3 s were identified as strabismus.

3. Strabismic amblyopia

Amblyopia manifests as an ocular misalignment in the absence of refractive errors that could be specified for combined-mechanism amblyopia.

4. Anisometropic amblyopia

Anisometropic amblyopia occurs when an uncorrected refractive error difference exists. The diopter difference of both eyes is greater than − 3.00 D for myopia, greater than or equal to + 1.00 D for hyperopia, and greater than ± 1.50 D for astigmatism.

5. Mixed amblyopia

Amblyopia is caused by both strabismus and refractive errors.

### Statistical analyses

Statistical analysis was performed using SPSS (version 27.0: IBM, Armonk, NY, USA) and presented using GraphPad Prism (GraphPad Software, San Diego, CA, USA). Descriptive statistics were shown in number and percentages. The prevalence was calculated as a percentage of the total population. Categorical data were evaluated using Fisher’s exact test. Continuous variables were presented as mean and standard deviation (mean ± SD) and calculated with a Student *t*-test. Multivariate linear regression was used to analyse the association between BCVA and age in children. *P* < 0.05 was considered statistically significant.

## Results

### Demographic characteristics of participants

Participants with an average age of 6.91 ± 2.46 years old were enrolled in our study. Among them, 30 (3.26%) were identified as having strabismus, 129 (14.02%) were found with unilateral amblyopia, and 94 (10.22%) were diagnosed as bilateral amblyopia. No significant sex differences were observed between children with and without strabismus or amblyopia (all *P* > 0.05). The main age range in each group was 5–10 yrs, followed by the age group < 5 yrs, as shown in Table 1.

**Table 1:** Demographic characteristics of participants

Classification	Strabismus		Amblyopia		
	With	Without	With		Without
			Unilateral Amblyopia	Bilateral Amblyopia	
Overall, n (%)	30	890	129	94	697
Gender, n (%)					
Girl	17 (56.67%)	461 (51.80%)	65 (50.39%)	45 (47.87%)	367 (47.20%)
Boy	13 (43.33%)	429 (48.20%)	64 (49.61%)	49 (52.13%)	329 (52.65%)
Age, n (%)					
<5 yrs	6 (20.0%)	150 (16.85%)	25 (19.38%)	21 (22.34%)	110 (15.78%)
5–10 yrs	19 (63.3%)	665 (74.72%)	98 (75.97%)	66 (70.21%)	520 (74.61%)
>10 yrs	5 (16.7%)	75 (8.43%)	6 (4.65%)	7 (7.45%)	67 (9.61%)

Distribution of clinical characteristics including age and gender in different groups. Data were presented in number and percentages

### Clinical characteristics of amblyopic children

As shown in Table 2, children diagnosed with amblyopia tended to visit the clinic at an earlier age compared to those without the condition, although this disparity was not significant. This is probably due to poor vision (0.50 ± 0.22 vs. 0.87 ± 0.19, 0.53 ± 0.21 vs. 0.90 ± 0.16). Unilateral amblyopia (*n* = 129) was more common than bilateral amblyopia (*n* = 94), and children with unilateral amblyopia visited the clinic at a slightly older age (6.84 ± 2.58 vs. 6.50 ± 2.43). A larger SE and shorter AL were observed in patients with amblyopia (3.16 ± 3.66, 2.96 ± 4.17 vs. 0.82 ± 2.37, *P* < 0.0001, 21.86 ± 1.50, 21.69 ± 1.58 vs. 22.84 ± 1.29, *P* < 0.0001).

**Table 2:** Clinical profile of amblyopia

Characteristics	Unilateral Amblyopia (*n* = 129)			Bilateral Amblyopia (*n* = 94)	Without (*n* = 697)	*P* Fellow vs. Uni Amblyopia	*P* Fellow vs. Without	*P* Uni Amblyopia vs. Without	*P* Bi Amblyopia vs. Without	*P* Uni Amblyopia vs. Bi Amblyopia
	Fellow		Amblyopia							
Age, yrs	6.84 ± 2.58	6.84 ± 2.58		6.50 ± 2.43	6.98 ± 2.43	/	0.5518	0.5518	0.0726	0.3205
VA, decimal	0.87 ± 0.19	0.50 ± 0.22		0.53 ± 0.21	0.90 ± 0.16	**< 0.0001**	0.0582	**< 0.0001**	**< 0.0001**	0.3065
SE, D	1.88 ± 2.51	3.16 ± 3.66		2.96 ± 4.17	0.82 ± 2.37	**0.0012**	**< 0.0001**	**< 0.0001**	**< 0.0001**	0.7044
AL, mm	22.39 ± 1.29	21.86 ± 1.50		21.69 ± 1.58	22.84 ± 1.29	**0.0026**	**0.0003**	**< 0.0001**	**< 0.0001**	0.4147

Distribution and comparison of age and ocular parameters including VA, SE, and AL in different categories. Bold font data indicate *P* < 0.05

VA, visual acuity; SE, spherical equivalent; AL, axial length; D, diopter

### Category of amblyopic children

The causes of unilateral and bilateral amblyopia were further analysed (Table 3). The anisometropic type (89, 68.99%) was the most common type of unilateral amblyopia. High hyperopia (50, 38.76%; 75, 39.89%) was most frequently observed in both amblyopia groups. Three children had unilateral amblyopia and strabismus, whereas four presented with bilateral amblyopia and strabismus.

**Table 3:** Proportion of amblyopia by different causes. To present the proportion, number and frequency were employed

Category	Amblyopia	
	Unilateral	Bilateral
Mild hyperopia, eyes (n,%)	7 (5.43%)	16 (17.02%)
Moderate hyperopia, eyes (n,%)	49 (37.98%)	52 (55.32%)
High hyperopia, eyes (n,%)	50 (38.76%)	75 (79.79%)
Mild myopia, eyes (n,%)	6 (4.65%)	18 (19.15%)
Moderate myopia, eyes (n,%)	3 (2.33%)	9 (9.57%)
High myopia, eyes (n,%)	3 (2.33%)	3 (3.19%)
Mixed, (n,%)	3 (2.33%)	4 (4.26%)
Anisometropic, (n,%)	89 (68.99%)	29 (30.85%)

### Clinical characteristics of strabismus children

In our study, 30 children were identified as having strabismus. The patients visited our department at an average age of 7.37 ± 2.88 years. No significant differences were identified between the groups with or without strabismus in the VA, SE and AL (Supplementary Table 1).

### Association between BCVA and age in amblyopia children

We further explored whether the BCVA in both groups correlated with the age at which they visited the hospital. After adjusting for sex, SE and AL, the multiple regression analysis suggested a positive correlation between age and BCVA (β = 0.621, 0.370, *P*<0.001) (Table 4).

**Table 4:** Multivariate analysis for BCVA in two types of amblyopia patients

	β	*P*	*R* ^2^	F
Bilateral Amblyopia	0.621	**<0.001**	0.448	24.232
Unilateral Amblyopia	0.370	**<0.001**	0.223	5.923

Adjusted for sex, SE and AL in a multivariate logistic regression model. Bold font data indicate *P* < 0.05

SE, spherical equivalent; AL, axial length

### Prevalence of refractive error

Overall, eyes with emmetropia (494, 36.65%) constituted the largest group, followed by those with moderate hyperopia (305, 22.63%) and mild myopia (277, 20.55%). High (3, 0.22%) and moderate myopia (18, 1.34%) were rare in our cohort. The distribution of the refractive status in boys and girls was similar, except that no girls presented with high myopia, whereas 3 (0.47%) boys developed high myopia (Table 5).

As shown in Supplementary Fig. 1, refractive status varied significantly at different age intervals. The prevalence of high hyperopia gradually tapered from 27.42% at 3-year-old to 8.74% at 6-year-old. Mild myopia rose from 1.61 to 12.62% at 6-year-old and 21.05% at 7-year-old. Subsequently the number increased with age.

**Table 5:** Proportion of hyperopia, emmetropia, and myopia in eyes without amblyopia or strabismus. To present the proportion, number and frequency were employed

Category	Boy *n* = 319	Girl *n* = 354	All *n* = 674
Mild hyperopia, eyes (n,%)	82 (12.85%)	66 (9.32%)	148 (10.98%)
Moderate hyperopia, eyes (n,%)	134 (21.00%)	171 (24.15%)	305 (22.63%)
High hyperopia, eyes (n,%)	50 (7.84%)	53 (7.49%)	103 (7.64%)
Emmetropia, eyes (n,%)	236 (36.99%)	257 (36.30%)	494 (36.65%)
Mild myopia, eyes (n,%)	120 (18.81%)	156 (22.03%)	277 (20.55%)
Moderate myopia, eyes (n,%)	13 (2.04%)	5 (0.71%)	18 (1.34%)
High myopia, eyes (n,%)	3 (0.47%)	0	3 (0.22%)

## Discussion

### Prevalence and causes of amblyopia

We screened 920 children in Shanghai, China in this hospital-based cross-sectional study. The average age at which the children visited the hospital and were diagnosed with amblyopia was 6.84 ± 2.58 and 6.50 ± 2.43 years. The reason might be that children went to school and carried increasing intensive schooling at 6–7-year intervals. During this period, they could meet with the difficulty in reading the blackboard clearly and then visited hospital. Besides, routine physical screening for freshman provided in some schools also helps identifying potential VA issues. A relatively high prevalence of amblyopia (24.24%) was identified in the present population, compared with the common school-based assessment. 129 children had unilateral amblyopia, and 94 were bilateral. The reported prevalence of amblyopia was 1.43% in a total of 9,263 multiethnic school-aged children in rural China [6], 1.2% in a total of 5667 preschool children in eastern China [7], and 1.0% in a total of 2,893 year 1 primary school students in central China [8]. Higher prevalence was also observed in other hospital-based studies worldwide, ranging from 6.8 to 72.9% (Supplementary Table 2). This discrepancy in prevalence is probably attributable to the different study properties, populations and regional economics. Notably, Gebru et al. and Agaje et al. indicated a high prevalence rate of 23.80% and 16.7%, respectively, which was similar with that in our current survey [8, 9]. To our knowledge, no hospital-based studies on amblyopia were conducted in Shanghai, China, therefore, no comparison could be made. As an economic centre in China, school-based vision screening programs are more widely promoted in Shanghai, therefore, most children enrolled in our study are admitted to the hospital after the screening. This might causes a high rate of amblyopia in our study. In addition, better parental consciousness about routine visual examination; better public education and web-based health-care promotion all help referring more potential patients to hospitals.

In terms of the causes of amblyopia, high hyperopia accounted for the highest risk factor, and moderate hyperopia ranked second, regardless of the amblyopia type. The anisometropic type was the most common for unilateral amblyopia, accounting for the entire cohort, occupying 68.99% of the total cohort. 3 (2.33%) children presented with both unilateral amblyopia and strabismus. This rate was similar to the previous report in the Anyang Childhood Eye Study, in which 72.22% were anisometropic amblyopia. Similarly, a significant refractive error was observed in patients with bilateral amblyopia in the Anyang cohort [10]. Our study and previous reports imply a high risk of amblyopia in children with significant refractive errors. These findings could guide ophthalmologists to better understand the clinical profile of amblyopia, as well as suggest early screening for refractive errors.

The multiple regression analysis indicated that older age was associated with better BCVA in the amblyopia cohort. This was probably because when children did not complain of decreased vision, their parents would not know about their situation and take them to seek health care. Therefore, children who visited the hospital later usually had a relatively better VA.

In summary, the high prevalence of amblyopia in our study revealed the necessity to prompt early screening in school-age individuals. Early detection and access to treatment would be beneficial for amblyopia prognosis. In addition, high hyperopia was the leading cause of amblyopia, therefore, timely treatment such as glasses was recommended.

### Prevalence of strabismus

In our study, 30 (3.26%) children were identified as having strabismus. This rate is similar to that reported in population-based studies conducted in China (Supplementary Table 3). The same study group reported a 5.65% and 5.56% prevalence among preschool-age children in the Nanjing area in 2015 and 2021. Another study in Mojiang Hani Autonomous County found a lower prevalence (1.93%) in schooling children [9]. Our data were intermediate, probably because of the wide age range in which we enrolled.

### Refractive error

In the present study, the prevalence of mild, moderate, and high hyperopia was 10.98%, 22.63% and 7.64% in the cohort with normal VA, which was in line with the reported prevalence rates in the prior studies. Children at about 6 years old were mostly mildly hyperopic [11] (Supplementary Table 4). When further subdivided by age, the prevalence of high hyperopia showed a descending tendency with an increase in age, gradually decreasing from 27.42% at 3-year-old to 8.74% at 6-year-old (Supplementary Fig. 1). Instead, mild myopia increased dramatically from 1.61 to 12.62% at 6-year-old and 21.05% at 7-year-old. This result was similar to study by Cheng et al. who reported a 25.5% rate [12]. This rapid growth pattern was probably caused by educational pressure and homework demands, as 6 and 7-year-olds are the first and second primary school years in China. Our current study included a high proportion (64.69%) of children aged 3–5 years, thereby, elucidating the influence of school exposure on refractive development, apart from physiological emmetropisation. There was a high percentage of different types of refractive errors in these young subjects, suggesting the necessity of public attention on refractive error issue, especially in children reaching the crossroads between preschool and school. Furthermore, larger population-based studies on prevalence and pattern of refractive errors are also urgent to validate and expand our findings.

### Limitations

This study had some limitations. First, the age was not uniformly distributed in our study. Most children were between 5 and 10 years old, which might cause some bias in results, especially in the refrative status analyses. Second, our analysis did not include potential risk factors such as parental myopia, which might lead to omissions in analyzing the potential causes. Further evaluations of more potential risk factors are advisable to clarify the causes of amblyopia and refractive error. Thirdly, a limited sample of strabismus were identified in our study, therefore, we were ubable to divide them into various groups and perform further comparison. Larger populations are necessary to broaden our data. Finally, as this was a cross-sectional study, age-related changes could not be observed: therefore, longitudinal studies with a longer follow-up are urgrntly needed to establish the age-related profile of refractive status, as well ass demonstrate the cause-and-effect relationship between risk factors and amblyopia.

## Conclusion

This hospital-based cross-sectional study investigated the prevalence and clinical profiles of amblyopia and strabismus among children and adolescents aged 3–16 years in Shanghai, China. We also analysed the refractive distribution of the children at each age interval. Our findings facilitate future longitudinal studies targeting age-related changes in amblyopia, strabismus, and refractive error. Due to the high prevalence of amblyopia and refractive errors, timely screening and management are urgent, especially for children reaching school age.

### Electronic supplementary material

Below is the link to the electronic supplementary material.

Supplementary Material 1

## Data Availability

All data generated or analyzed during this study are included in this published article. Further enquiries can be directed to the corresponding author.
