# Supplementary material for: Prevalence and characteristics of amblyopia, strabismus, and refractive errors among patients aged 3–16 years in Shanghai, China: a hospital-based population study
Source: BMC Ophthalmol. 2024 Jun 7;24:239. doi: 10.1186/s12886-024-03477-8 (PMC11157824; doi:10.1186/s12886-024-03477-8)
Supplement: Supplementary file 1 — Supplementary Material 1 [file 12886_2024_3477_MOESM1_ESM.docx]

| **Classification** | **Strabismus** | | |
| --- | --- | --- | --- |
|  | **With**  **(n=30)** | **Without**  **(n=890)** | ***P* value** |
| Age, yrs | 7.37±2.88 | 6.90±2.44 | 0.3027 |
| VA,decimal | 0.82±0.24 | 0.83±0.22 | 0.8072 |
| SE, D | 1.55±2.57 | 1.26±2.86 | 0.5839 |
| AL,mm | 22.72±1.29 | 22.64±1.39 | 0.7561 |

Supplementary Table 1: Clinical profile of strabismus.

Distribution of clinical characteristics including age, VA, SE, and AL in individuals with or without strabismus.

VA, visual acuity; SE, spherical equivalent; AL, axial length; D, diopter.

| **Participants** | **Age** | **Prevalence** | **Area** | **Publication date** | **Reference** |
| --- | --- | --- | --- | --- | --- |
| 107 | 4‒12 | Refractive amblyopia: 72.9% | Gaza Ophthalmic Hospital, Gaza Strip | 2021 | Eslayeh et al. 2021 |
| 1038 | 3-16 | Amblyopia: 6.80% | Eye Institute, Harare, Zimbabwe | 2022 | Kwarteng et al. 2022 |
| 202 | 5-15 | Amblyopia: 23.80% | Hawassa university comprehensive specialized hospital | 2022 | Gebru et al. 2022 |
| 582 | / | Strabismus: 17.9%;  Amblyopia: 16.7% | Hawassa University Comprehensive Specialized Hospital | 2020 | Agaje et al. 2020 |
| 1350 | 1–15 | Strabismus: 38%;  Amblyopia: 9.1% | Private Hospital in Dammam, Kingdom of Saudi Arabia | 2015 | Al-Tamimi et al. 2015 |
| 3337 | 0-3 | Strabismic amblyopia: 6.0% | An Australian tertiary paediatric hospital | 2023 | Lo-Caoet al. 2023 |

Supplementary Table 2: Hospital-based studies about prevalence of amblyopia and strabismus in children. Data including participant number, age, prevalence, area, and publication year were collected.

| **Participants** | **Age** | **Prevalence** | **Area** | **Publication date** | **Reference** |
| --- | --- | --- | --- | --- | --- |
| 5667 | 3-6 | Amblyopia:1.2%;  Strabismus: 5.65%. | Yuhua District, Nanjing | 2015 | Chen et al. 2016 |
| 1961 | 4-5 | Strabismus: 5.56%. | Yuhua District, Nanjing | 2021 | Wang et al. 2021 |
| 3050 | Grade  1 and 7 | Amblyopia: 0.82%;  Strabismus: 1.93%. | Mojiang Hani Autonomous County | 2019 | Zhu et al. 2019 |

Supplementary Table 3: Studies about prevalence of amblyopia and strabismus among children conducted in China. Data including participant number, age, prevalence, area, and publication year were collected.

| **Participants** | **Age** | **Prevalence** | **Amblyopia/**  **Strabismus result** | **Area** | **Publication date** | **Reference** |
| --- | --- | --- | --- | --- | --- | --- |
| 9512 | 7-12 | The prevalence of UCVA, presenting, and BCVA of 20/40 or worse in the better eye were 13.33%, 11.26%, and 0.63%. | Amblyopia accounted for 10.12% of VI. | Shanghai | 2014 | He et al. 2014 |
| 8267 | 3-10 | The prevalence of UCVA, presenting visual acuity, and BCVA in the better eye of ≤20/40 was 19.8%, 15.5%, and 1.7% | Amblyopia accounted for 0.93% of VI. | Jiading District, Shanghai | 2016 | Ma et al. 2016 |
| 162 | 3-20 | The prevalence of moderate VI, severe VI, and blind was 43.21%, 26.54%, and 19.75%. | / | Low vision clinic of the Eye and ENT Hospital,Shanghai | 2016 | Gao et al. 2016 |
| 7166 | 4-6 | The prevalence of myopia, hyperopia, and astigmatism was 5.9%, 1.0%, and 12.7% | / | Shanghai | 2019 | Li et al. 2019 |
| 1722 | 7-9 | The prevalence of myopia was 25.6%. | / | Pudong New Area, Shanghai | 2023 | Cheng et al. 2023 |
| 7084 | 4-15 | The prevalence of (RA)/CA in children was 15.8%/64% in kindergartens, 16.5%/65% in primary schools, and 32.8%/76.9% in junior high schools. | / | Minhang District, Shanghai | 2023 | Zhang et al. 2023 |
| 2851 | 3-6 | The prevalence of myopia and astigmatism was 3.7% and 18.3%. | / | Jiading and Xuhui District, Shanghai | 2018 | Zhang et al. 2018 |
| 606 476 | 4-14 | The prevalence of myopia and high myopia was 32.9% and 4.2%. | / | All 17 districts, Shanghai | 2021 | He et al. 2021 |
| 1923 | Grade  1-4 | The prevalence of myopia was 7.42% for residents and 8.15% for migrants for children in grade one. | / | Baoshan District, Shanghai | 2018 | Ma et al. 2018 |

Supplementary Table 4: Refractive error and visual impairment prevalence among children in Shanghai. Data including participant number, age, prevalence, area, and publication year were summarized. If amblyopia or strabismus results were available, we also collected them.

UCVA, uncorrected visual acuity; BCVA, best-corrected visual acuity; RA, refractive astigmatism; CA, corneal astigmatism.


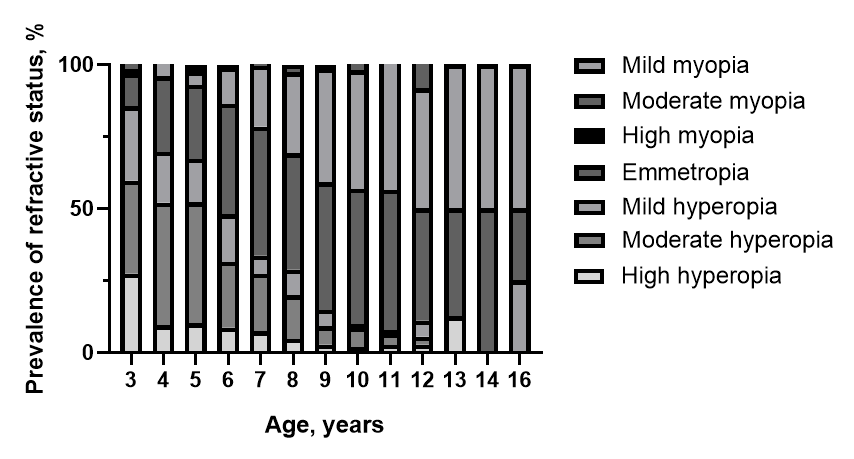


Supplementary Figure 1: Age-specific distribution of refractive status in eyes without amblyopia or strabismus. Refractive status including mild myopia, moderate myopia, high myopia, emmetropia, mild hyperopia, moderate hyperopia, and high hyperopia were analyzed. Data were shown in percentage (%).
